# [^3^H]-NFPS binding to the glycine transporter 1 in the hemi-parkinsonian rat brain

**DOI:** 10.1007/s00221-024-06815-w

**Published:** 2024-03-25

**Authors:** Imane Frouni, Esther Kim, Judy Shaqfah, Dominique Bédard, Cynthia Kwan, Sébastien Belliveau, Philippe Huot

**Affiliations:** 1 Département de Pharmacologie et Physiologie, Université de Montréal, Montreal, QC Canada; 2 Neurodegenerative Disease Group, Montreal Neurological Institute-Hospital (The Neuro), 3801 University St, Montreal, QC H3A 2B4 Canada; 3 Department of Neurology and Neurosurgery, McGill University, Montreal, QC Canada; 4 Movement Disorder Clinic, Division of Neurology, Department of Neurosciences, McGill University Health Centre, Montreal, QC Canada

**Keywords:** GlyT1, Parkinson’s disease, Dyskinesia, Org-25,935, [^3^H]-NFPS, Autoradiography, 6-OHDA-lesioned rat

## Abstract

L-3,4-dihydroxyphenylalanine (L-DOPA) is the main treatment for Parkinson’s disease (PD) but with long term administration, motor complications such as dyskinesia are induced. Glycine transporter 1 (GlyT1) inhibition was shown to produce an anti-dyskinetic effect in parkinsonian rats and primates, coupled with an improvement in the anti-parkinsonian action of L-DOPA. The expression of GlyT1 in the brain in the dyskinetic state remains to be investigated. Here, we quantified the levels of GlyT1 across different brain regions using [^3^H]-NFPS in the presence of Org-25,935. Brain sections were chosen from sham-lesioned rats, L-DOPA-naïve 6-hydroxydopamine (6-OHDA)-lesioned rats and 6-OHDA-lesioned rats exhibiting mild or severe abnormal involuntary movements (AIMs). [^3^H]-NFPS binding decreased in the ipsilateral and contralateral thalamus, by 28% and 41%, in 6-OHDA-lesioned rats with severe AIMs compared to sham-lesioned animals (*P* < 0.01 and 0.001). [^3^H]-NFPS binding increased by 21% in the ipsilateral substantia nigra of 6-OHDA-lesioned rats with severe AIMs compared to 6-OHDA-lesioned rats with mild AIMs (*P* < 0.05). [^3^H]-NFPS binding was lower by 19% in the contralateral primary motor cortex and by 20% in the contralateral subthalamic nucleus of 6-OHDA-lesioned rats with mild AIMs animals compared to rats with severe AIMs (both *P* < 0.05). The severity of AIMs scores positively correlated with [^3^H]-NFPS binding in the ipsilateral substantia nigra (*P* < 0.05), ipsilateral entopeduncular nucleus (*P* < 0.05) and contralateral primary motor cortex (*P* < 0.05). These data provide an anatomical basis to explain the efficacy of GlyT1 inhibitors in dyskinesia in PD.

**Supplementary Information:**

The online version contains supplementary material available at 10.1007/s00221-024-06815-w.

## Introduction

An important neuropathological feature of Parkinson’s disease (PD) is the loss of substantia nigra (SN) pars compacta (SNc) dopaminergic neurons, which results in reduced dopamine innervation in the striatum and motor dysfunction, notably bradykinesia (Hornykiewicz and Kish 1987). Accordingly, pharmacological approaches for PD have mostly centred on dopamine replacement, mainly with L-3,4-dihydroxyphenylalanine (levodopa, L-DOPA), which is successful at alleviating the motor symptoms of the condition (Fox et al. 2018). However, as the disease progresses, L-DOPA retains its efficacy but induces complications such as dyskinesia (Fox and Lang 2008). Up to 95% of patients with advanced PD experience dyskinesia which negatively impacts their quality of life (Hely et al. 2005).

Previously, a study reported that (S)-2-amino-6-chloro-N-(1-(4-phenyl-1-(propylsulfonyl)piperidin-4-yl)ethyl)benzamide (ACPPB), a glycine transporter 1 (GlyT1) inhibitor, enhanced functional dopaminergic reinnervation of the 6-hydroxydopamine (6-OHDA)-lesioned mouse dorsal striatum (Schmitz et al. 2013), suggesting a possible neuro-protective role of GlyT1 inhibition in PD. More recently, we have shown that GlyT1 inhibition might also be a potential therapeutic option for treatment-related complications and we have discovered that bitopertin, a selective non-sarcosine-derived GlyT1 inhibitor, improves the severity of parkinsonism, attenuates the induction of dyskinesia and alleviates established dyskinesia in the 6-OHDA-lesioned rat (Frouni et al. 2022). We also showed that ALX-5407 (also known as (R)-(N-[3-(4’-fluorophenyl)-3-(4’-phenylphenoxy)propyl])sarcosine [(R)-NFPS]), a selective sarcosine-based GlyT1 inhibitor, reduced L-DOPA-induced dyskinesia and improved parkinsonian disability in the 1-methyl-4-phenyl-1,2,3,6-tetrahydropyridine (MPTP)-lesioned marmoset (Frouni et al. 2021). Collectively, these studies indicate that GlyT1 inhibition might be a promising therapeutic strategy for PD and L-DOPA-induced dyskinesia.

The neuroanatomical distribution of GlyT1 has been extensively studied in healthy rodents. In the rat, GlyT1 mRNA and proteins were shown to be highly expressed in glial cells from the hypothalamus, thalamus, neocortex, retina and olfactory bulb, and were also found to be abundant in areas associated with glutamatergic terminals such as the hippocampus (Zafra et al. 1995; Cubelos et al. 2005). Similar results were obtained in multiple autoradiographic binding experiments, with high binding observed in the thalamus, cerebral cortical white matter and moderate binding in the hippocampus and the neocortical grey matter in rodents (Zeng et al. 2008; Hoffmann et al. 2021), non-human primates (Zeng et al. 2008; Borroni et al. 2013) and humans (Wong et al. 2013). In contrast to the normal physiological state, to the best of our knowledge, GlyT1 expression has not been studied in PD and L-DOPA-induced dyskinesia. In the present study, we sought to determine GlyT1 distribution in brain areas implicated in PD and L-DOPA-induced dyskinesia, i.e. the structures of the motor loop of the cortex – basal ganglia – thalamus – cortex circuit, by performing autoradiographic binding with [^3^H]-NFPS in sham-lesioned rats and 6-OHDA-lesioned rats that were treated with vehicle or L-DOPA and developed dyskinesia of variable severity.

## Materials and methods

### Animals

Experiments were carried out on adult female Sprague-Dawley rats (*N* = 31, weighing between 225 and 250 g, Charles River Laboratories, Saint-Constant, QC, Canada). Rats were housed in groups of three under controlled conditions of temperature (21 ± 1 °C), humidity (55%), and lighting (12-hour light/dark cycle, with lights turned on at 07:00). Rats had unrestricted access to food and water. Before the experiments, rats were left undisturbed for a minimum of 5 days to acclimate to their housing environment. All procedures were granted approval by the Animal Care Committee (Animal Use Protocol #2017–7922) at the Montreal Neurological Institute-Hospital (The Neuro), following guidelines established by the Canadian Council on Animal Care.

### Induction of hemi-parkinsonism

Hemi-parkinsonism was induced by unilateral injection of 6-OHDA, as previously described (Frouni et al. 2019, 2022; Hamadjida et al. 2020). Initially, animals were administered desipramine (10 mg/kg subcutaneously [s.c.], MilliporeSigma, Oakville, ON, Canada) and pargyline (5 mg/kg s.c., MilliporeSigma) to prevent noradrenergic neuron damage (Ungerstedt 1968). Rats were anaesthetised using isoflurane (2–4%) in 100% oxygen (1 L/min) and positioned in a stereotaxic frame (David Kopf Instruments, Tujunga, CA, USA). After a period of 30 min, a unilateral injection of 6-OHDA hydrobromide (7 µg/µL, MilliporeSigma) was performed in the right medial forebrain bundle and the injection site coordinates relative to Bregma were as follows: antero-posterior: −2.8 mm, medio-lateral: −2.0 mm, dorso-ventral: −9.0 mm (Paxinos and Watson 2007). The incisor bar was set 3.3 mm below the ear bars. For comparison, sham-lesioned animals received an injection of 6-OHDA vehicle (0.9% saline with 0.02% ascorbate) using the same coordinates mentioned above.

### Assessment of hemi-parkinsonism

After a three-week recovery period, animals underwent the cylinder test to evaluate the extent of hemi-parkinsonism, by counting the number of rears of both forepaws, separately and concomitantly, on a cylinder wall (Schallert et al. 2000; Frouni et al. 2022). Rats were placed in a transparent cylinder (14 cm diameter × 28 cm height). Rats were recorded for a duration of 10 min and their behaviour was analysed *post hoc*. Only animals that used the forepaw ipsilateral to the lesion side (the non-parkinsonian forepaw) in at least 70% of rearing were chosen for further investigation. This criterion represents a score indicative of a striatal dopamine deficit greater than 88% (Schallert et al. 2000).

### Experimental groups

Rats that met the inclusion threshold after the cylinder test were divided into four distinct groups: (1) sham group (vehicle-lesioned, treated with vehicle [0.1% ascorbate in 0.9% NaCl], *N* = 8), (2) parkinsonian group (6-OHDA-lesioned, treated with vehicle, i.e. not exposed to L-DOPA, *N* = 8), (3) mildly dyskinetic group (6-OHDA-lesioned, treated with L-DOPA and exhibiting mild dyskinesia, *N* = 7), (4) severely dyskinetic group (6-OHDA-lesioned, treated with L-DOPA and exhibiting severe dyskinesia, *N* = 9). Vehicle and L-DOPA treatments were administered once daily s.c. for 14 days. L-DOPA was administered as L-DOPA/benserazide 10/15 mg/kg.

### Assessment of abnormal involuntary movements severity

After the 14-day dyskinesia induction period, L-DOPA-treated 6-OHDA-lesioned rats were injected with L-DOPA (10/15 mg/kg, s.c.) and the severity of axial torsion, limb movements, oro-lingual stereotypies (ALO) abnormal involuntary movements (AIMs) was evaluated by an experienced observer blinded to the treatment, according to a scale that evaluates severity-based, i.e. “amplitude” and time-based, i.e. “duration” AIMs (Cenci and Lundblad 2007). ALO AIMs were assessed for a duration of 2 min every 20 min, for a total observation period of 180 min, plus a baseline assessment. In summary, one experimental session lasted 3 h. During these 3 h, we assess the severity of AIMs every 20 min. Each of these assessments last 2 min. The duration and amplitude of the individual ALO components were individually rated on a scale ranging from 0 to 4 for each monitoring interval, with a maximum obtainable score of 36 per session. The “integrated” ALO AIMs score was defined as the product of ALO AIMs duration × ALO AIMs amplitude, as previously described (Frouni et al. 2018).

### Tissue preparation

Animals were administered their regular L-DOPA or vehicle treatment and, 45 min later, were euthanised by isoflurane overdose (2–4%) and subjected to trans-cardial perfusion with ice-cold 0.9% saline. Brains were collected and flash-frozen in isopentane at -56 °C and stored at -80 °C until cryostat sectioning. Brains were set in optimal cutting temperature (OCT) in a cryostat at -20 °C (Leica CM3050 S; Leica Microsystems, Richmond Hill, ON, Canada) and sliced coronally into 12-µm thick sections. Sections were thaw-mounted on SuperFrost® Plus slides (Thermo Fisher Scientific, Mississauga, ON, Canada) and stored at -80 °C until use.

### Immunohistochemistry

Extent of lesion on striatal brain sections was examined by immunohistochemistry using a mouse monoclonal antibody raised against tyrosine hydroxylase (TH) (1:1000, MilliporeSigma, #MAB318) as previously described (Kwan et al. 2022). Frozen striatal brain sections mounted on slides were air-dried overnight at room temperature and then fixed by immersing them in pre-cooled acetone (-20 °C) for 10 minutes, followed by a 20-minute air-drying period. Sections were immersed in 0.5% H_2_O_2_ for 10 minutes to block endogenous peroxidase activity. Thereafter, sections were incubated for 1 h in 10% normal goat serum (NGS) and 5% bovine serum albumin (BSA) in Tris buffered saline (TBS; 100 mM Tris-Cl, pH 7.40, containing 240 mM NaCl) containing 0.3% Triton X-100 to block non-specific immunoreaction. Then, sections were incubated with the TH antibody in 5% NGS and 2% BSA in TBS containing 0.1% Triton X-100 (TBS-T) overnight at 4 °C. Sections were incubated in the presence of goat anti-mouse biotinylated secondary antibody (1:200, Invitrogen, Waltham, MA, USA, #31,800) in 5% NGS and 2% BSA in TBS-T for 1 h, followed by an incubation in avidin-biotin complex detection kit (ABC; Vector Laboratories, Newark, CA, USA, #PK-6100) for 2 h. The immunoreaction in sections was visualised in TBS-T containing 1.25 mg/mL nickel ammonium sulphate hexahydrate (MilliporeSigma, #574,988), 0.25 mg/mL 3,3’-diaminobenzidine (MilliporeSigma, #D5637) and 0.015% H_2_O_2_. Finally, sections were dried, rehydrated in water, dehydrated in graded alcohol solutions, cleared with xylene and coverslipped using Permount mounting medium (Thermo Fisher Scientific, SP15-100).

TH-immunoreactivity levels were assessed using densitometry in sections encompassing the dorsolateral striatum (Bregma ∼ +1.20 mm). Images were captured by a Nikon Eclipse E800 microscope (The Neuro Microscope Core Facility) using Stereo Investigator software (MBF Bioscience, Williston, VT, USA, version 11). The optical density in both ipsilateral and contralateral dorsolateral striata was measured across four adjacent brain sections using ImageJ software (NIH, Bethesda, MD, USA, version 1.53c). The average relative TH optical density was computed for each side, on every animal.

### [^3^H]-NFPS autoradiographic binding

As mentioned in the Introduction, the regions of interest were selected for analysis based on their implication in the motor loop of the basal ganglia (McGregor and Nelson 2019) and were identified from a standard rat brain atlas (Paxinos and Watson 2007). The investigated areas consist of the primary motor cortex (M1), striatum (encompassing both the caudate nucleus and putamen, henceforth abbreviated as CPu), globus pallidus (GP), entopeduncular nucleus (EPN), subthalamic nucleus (STN), ventral anterior/ ventral lateral (VA/VL) nuclei of the thalamus, as well as the SN.

Four consecutive sections were processed for each brain area to determine total binding, and four to evaluate non-specific binding. Sections were thawed and dried at room temperature overnight. Sections were pre-incubated by washing twice in Krebs-HEPES buffer (50 mM, pH 7.4) for 15 min at room temperature and then incubated in Krebs-HEPES binding buffer (50 mM, pH 7.4) containing 10 nM [^3^H]-NFPS (American Radiolabeled Chemicals, St. Louis, MO, USA; specific activity: 59.72–60.23 Ci/mmol) for 60 min at room temperature to assess total binding. Non-specific binding was assessed by the addition of 1 µM Org-25,935 (Cedarlane Laboratories, Burlington, ON, Canada) to the Krebs-HEPES binding buffer containing 10 nM [^3^H]-NFPS. Sections were then washed three times in 4 ºC Krebs-HEPES buffer (50 mM, pH 7.4; 4ºC) for 10 min to end the binding. Following this, sections were briefly dipped in 4 ºC ddH_2_O and air dried at room temperature. Sections were opposed to [^3^H]-sensitive Biomax MR films (MilliporeSigma, Canada) for 3 weeks at room temperature, along with [^3^H]-microscale standards (ART0123B and ART0123C, 5 mm × 7 mm; American Radiolabeled Chemicals). Films were developed and autoradiograms were obtained for densitometric analysis.

Autoradiograms were analysed using optical densitometry with ImageJ software (version 1.52p). Beta-emitting [^3^H]-microscale standards of known radioactivity were employed to construct a reference curve that correlates radioactivity with the grey scale values observed in autoradiograms (Zilles et al. 2002). This curve was then used to quantify the intensity of the signal in nanoCuries (nCi) per mg of tissue. Background values were subtracted from total and non-specific binding values. Specific binding was calculated by subtracting non-specific binding values from total binding.

### Statistical analysis

Cylinder test data are presented as the mean ± standard error of the mean (SEM) and were analysed by one-way analysis of variance (ANOVA) followed by Tukey’s tests. Relative striatal TH optical density expression is presented as a percentage of the unlesioned hemisphere and were analysed using Student’s *t* test.

Time courses of ALO AIMs scores are presented as the median and were analysed by computing the area under the curve (AUC), followed by unpaired Welch’s unequal variances t-test.

For each region of interest, specific [^3^H]-NFPS binding levels are displayed as the mean ± SEM. Levels of binding for each region of each animal group were analysed by planned comparisons with multiple Student’s *t* tests and *P* values were corrected using the Holm-Sidak multiple comparisons test.

Correlation between PD severity and ALO AIMs scores with specific [^3^H]-NFPS binding were determined using the Pearson correlation coefficient.

For all analyses, statistical significance was assigned when *P* ˂ 0.05. Statistical analyses were performed with GraphPad Prism 8.4.3 (GraphPad Software, Boston, MA, USA).

## Results

### Parkinsonism and lesion severity

**Fig. 1:**
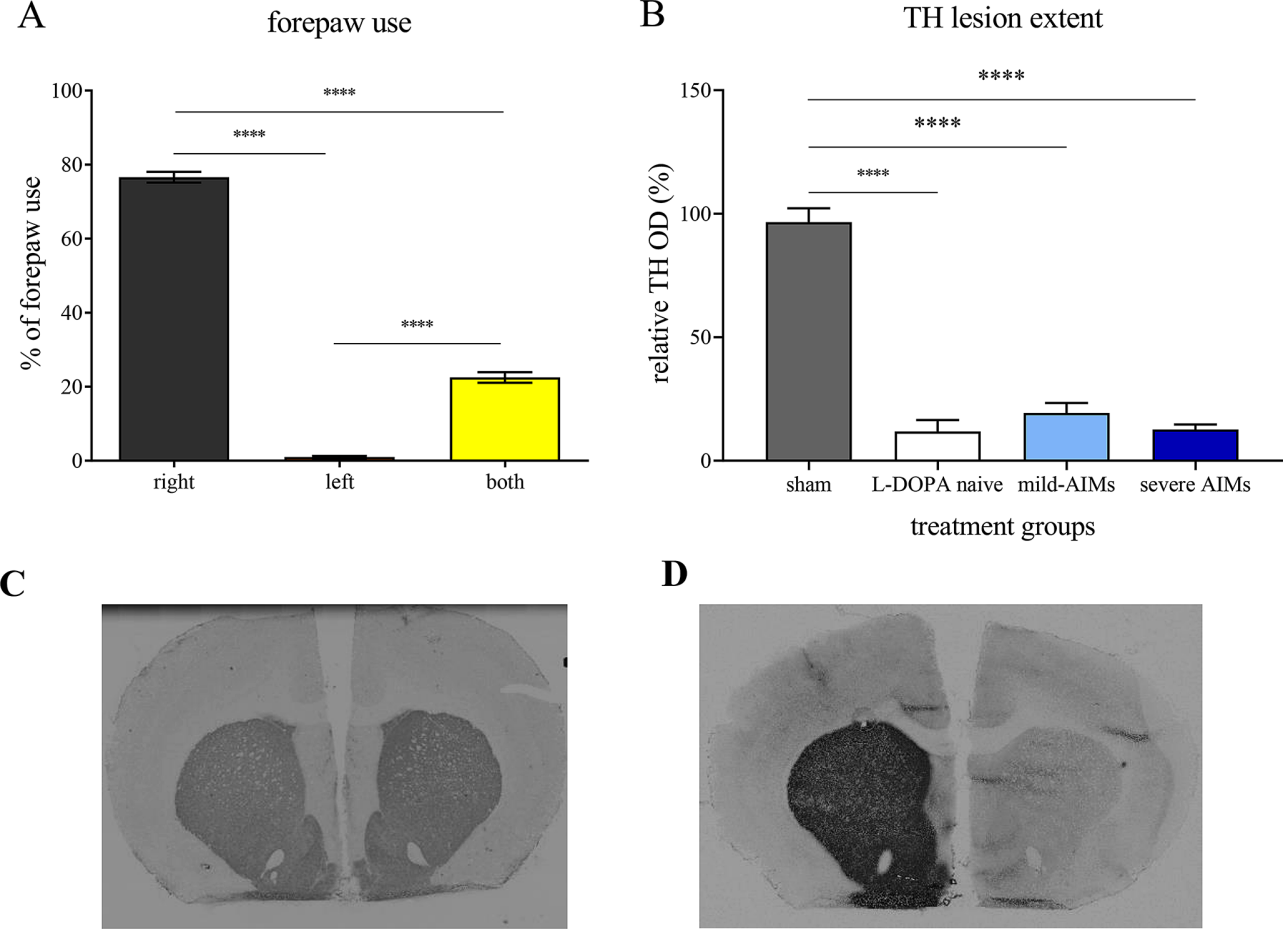
6-OHDA-lesioned rats (*N* = 24) selected for the autoradiographic binding experiment exhibited a significantly higher use of the parkinsonian (right) forepaw compared to the non-parkinsonian (left) forepaw and both forepaws. The rearing asymmetry was assessed during the cylinder test (**A**). Relative tyrosine hydroxylase (TH) optical density (OD) was significantly reduced in the dorsolateral caudate-putamen (CPu) ipsilateral to the injection of 6-OHDA compared to sham-lesioned animals (**B**), with representative photomicrographs of TH immunoreactive fibres in the CPu of sham-lesioned (**C**) and 6-OHDA-lesioned (**D**) rats. Cylinder test data are presented as the mean ± standard error of the mean (SEM). Relative optical density measurements are presented as a percentage of the contralateral (non-lesioned) hemisphere and are graphed as the mean ± SEM. AIMs: abnormal involuntary movements. *N* = 8 in the sham-lesioned vehicle group; *N* = 8 in the L-DOPA-naïve 6-OHDA-lesioned group; *N* = 7 in the 6-OHDA-lesioned rats with mild AIMs group; *N* = 9 in the 6-OHDA-lesioned rats with severe AIMs group. ****: *P* < 0.0001

6-OHDA-lesioned rats exhibited marked rearing asymmetry [F_(2, 69)_ = 1,065, *P* < 0.0001, one-way ANOVA; Fig. 1A], with preferential use of the right (non-parkinsonian) forepaw in 76.58 ± 1.46% of wall contacts, compared to 0.96 ± 0.32% with the left (parkinsonian) (*P* < 0.0001, Tukey’s test) forepaw and 22.50 ± 1.43% with both forepaws (*P* < 0.0001, Tukey’s test), respectively, assessed by the cylinder test.

Accordingly, as illustrated in Fig. 1B and D, 6-OHDA injection significantly reduced TH optical density in the CPu (F_(3,28)_ = 95.75, *P* < 0.0001, one-way ANOVA). Indeed, L-DOPA-naïve 6-OHDA-lesioned rat, 6-OHDA-lesioned rats with mild AIMs and 6-OHDA-lesioned rats with severe AIMs exhibited reduction of TH optical density of 88.18%, 80.68% and 87.38% (all *P* < 0.0001, Holm-Sidak test), respectively, compared to sham-lesioned rats. No significant differences between the different 6-OHDA-lesioned groups were found. As shown in Fig. 1C, striatal TH immunoreactivity in sham-lesioned rats was comparable between hemispheres.

### ALO AIMs severity

After chronic administration of vehicle or L-DOPA, there were significant differences in ALO AIMs severity between the groups. Thus, as expected, AIMs were undetectable in sham-lesioned rats and 6-OHDA-lesioned rats treated with vehicle. The integrated ALO AIMs score of 6-OHDA lesioned rats with mild AIMs was 598.6 ± 122.3, whereas the score of severely dyskinetic 6-OHDA-lesioned rats was 5,194.0 ± 765.7, which represents a ≈ 9-fold magnitude difference (*t*_(8.52)_ = 17.72, *P* < 0.0001).

### Specific [^3^H]-NFPS binding is altered in the brain of 6-OHDA-lesioned rats

**Fig. 2:**
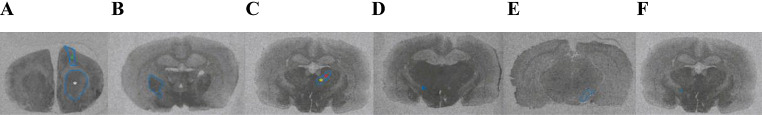
Representative autoradiograms of total [^3^H]-NFPS binding in the rat caudate-putamen (CPu, white *; **A**), motor cortex (M1, green *; **A**), globus pallidus (GP, **B**), ventral anterior (VA, red *, **C**)/ventral lateral (VL, yellow *, **C**) thalamus, subthalamic nucleus (STN, **D**), substantia nigra (SN, **E**) and entopeduncular nucleus (EPN, **F**) are presented. All autoradiograms were selected from sham-lesioned rats

**Fig. 3:**
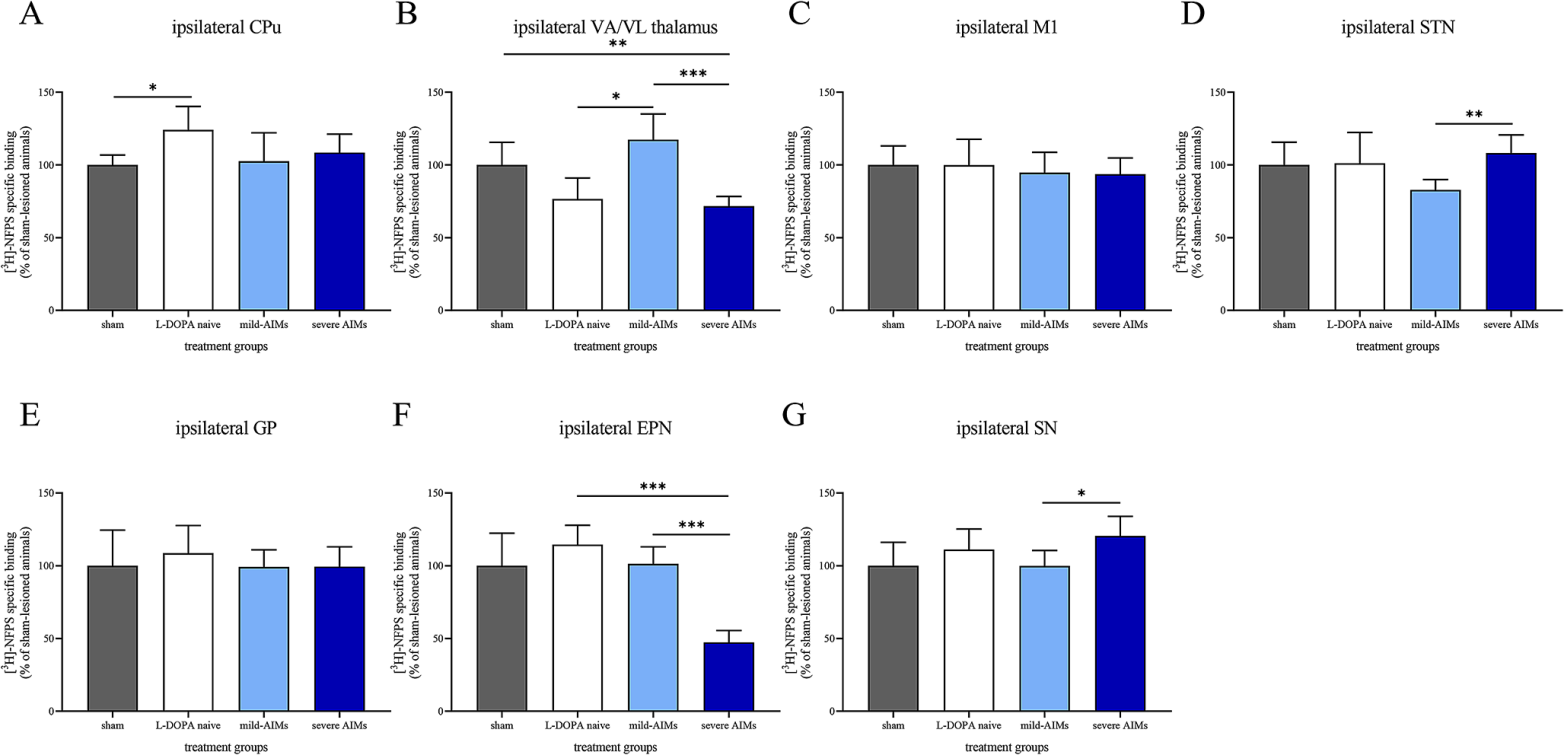
[^3^H]-NFPS binding levels across the examined brain areas ipsilateral to 6-OHDA injection. There was a 24% increase in [^3^H]-NFPS binding in the ipsilateral caudate-putamen (CPu) of L-DOPA-naïve 6-OHDA-lesioned rats when compared to sham-lesioned animals (**A**). In the ventral anterior/ ventral lateral (VA/VL) thalamus, [^3^H]-NFPS binding was reduced by 28% in 6-OHDA-lesioned rats with severe abnormal involuntary movements (AIMs), compared to sham-lesioned rats. Decreases in [^3^H]-NFPS binding were also observed, by 33% and 29%, when severely dyskinetic rats and L-DOPA-naïve 6-OHDA-lesioned rats were compared to mildly dyskinetic 6-OHDA-lesioned animals (**B**). [^3^H]-NFPS binding was comparable between all groups of 6-OHDA-lesioned rats and sham-lesioned animals in the primary motor cortex (M1, **C**). [^3^H]-NFPS binding was decreased by 23% in the ipsilateral subthalamic nucleus (STN) of 6-OHDA-lesioned rats with mild AIMs compared to 6-OHDA-lesioned rats with severe AIMs (**D**). [^3^H]-NFPS binding in the ipsilateral globus pallidus (GP) was comparable between all groups of 6-OHDA-lesioned animals and sham-lesioned rats (**E**). [^3^H]-NFPS binding in the ipsilateral entopeduncular nucleus (EPN) was lower in severely dyskinetic 6-OHDA-lesioned rats, by 53% and 59%, when compared to mildly dyskinetic 6-OHDA-lesioned animals and L-DOPA-naïve 6-OHDA-lesioned rats, respectively (**F**). [^3^H]-NFPS binding in the ipsilateral substantia nigra (SN) was higher in severely dyskinetic 6-OHDA-lesioned rats, by 21%, when compared to mildly dyskinetic 6-OHDA-lesioned animals (**G**). [^3^H]-NFPS binding of 6-OHDA-lesioned rats, regardless of their group, is presented as a percentage of the binding densities encountered in sham-lesioned animals. Data are presented as the mean ± standard error of the mean (SEM). *N* = 5–9 per region per group. *: *P* < 0.05; **: *P* < 0.01; ***: *P* < 0.001

**Fig. 4:**
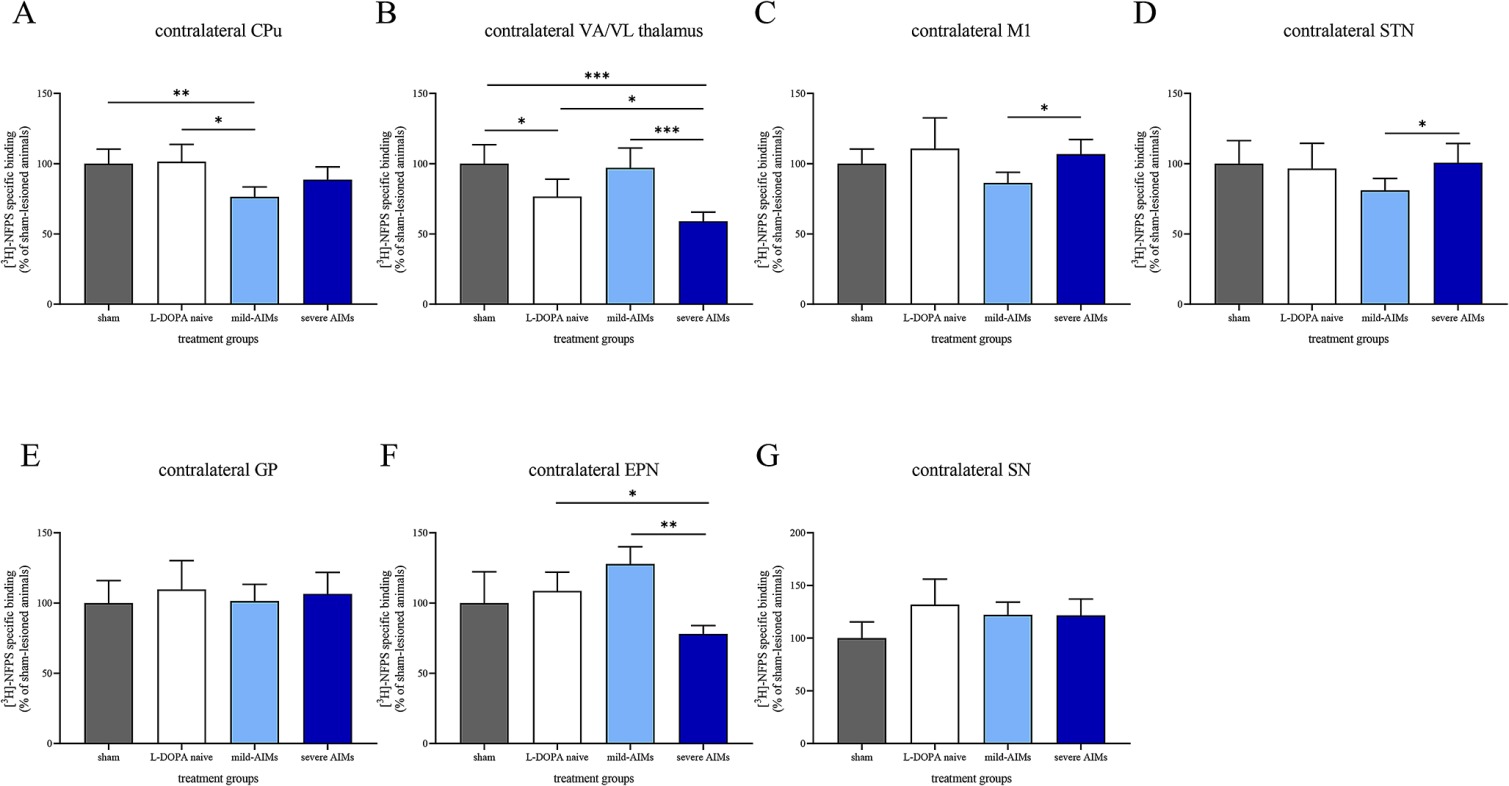
[^3^H]-NFPS binding levels across the examined brain areas contralateral to 6-OHDA injection. There was a 24% decrease in [^3^H]-NFPS binding in the contralateral caudate-putamen (CPu) of 6-OHDA-lesioned rats with mild abnormal involuntary movements (AIMs) when compared to both sham-lesioned and L-DOPA-naïve 6-OHDA-lesioned animals (**A**). In the contralateral ventral anterior/ ventral lateral (VA/VL) thalamus, [^3^H]-NFPS binding was decreased, by 41%, 23% and 34% when 6-OHDA-lesioned rats with severe AIMs were compared to sham-lesioned rats, L-DOPA-naïve 6-OHDA-lesioned rats and mildly dyskinetic 6-OHDA-lesioned rats, respectively. [^3^H]-NFPS binding in the contralateral VA/VL thalamus in L-DOPA-naïve 6-OHDA-lesioned rats was also diminished, by 23%, compared to sham-lesioned animals (**B**). [^3^H]-NFPS binding was reduced by 19% in the contralateral primary motor cortex (M1, **C**) and by 20% in the contralateral subthalamic nucleus (STN, **D**) when mildly dyskinetic 6-OHDA-lesioned rats were compared to 6-OHDA-lesioned rats with severe AIMs. [^3^H]-NFPS binding in the contralateral entopeduncular nucleus (EPN) was lower in severely dyskinetic 6-OHDA-lesioned rats, by 39% and 28%, when compared to mildly dyskinetic 6-OHDA-lesioned rats and L-DOPA-naïve 6-OHDA-lesioned rats, respectively (**F**). [^3^H]-NFPS binding in the contralateral globus pallidus (GP) and contralateral substantia nigra (SN) was comparable between all groups of 6-OHDA-lesioned animals and sham-lesioned rats (**E**, **G**). [^3^H]-NFPS binding of 6-OHDA-lesioned rats, regardless of their group, is presented as a percentage of the binding densities encountered in sham-lesioned animals. Data are presented as the mean ± standard error of the mean (SEM). *N* = 5–9 per region per group. *: *P* < 0.05; **: *P* < 0.01; ***: *P* < 0.001

Figure 2 shows examples of [^3^H]-NFPS binding autoradiograms. Figure 3 illustrates [^3^H]-NFPS binding levels across the examined brain areas ipsilateral to 6-OHDA injection, while Fig. 4 depicts [^3^H]-NFPS binding levels across the examined brain areas contralateral to 6-OHDA injection. Specific [^3^H]-NPFS binding levels in the ipsilateral hemisphere of brains of sham and 6-OHDA-lesioned rats are provided in Table 1, while specific [^3^H]-NFPS binding levels in the contralateral hemisphere of brains of normal and 6-OHDA-lesioned rats is detailed in Table 2. Table 3 presents, in a qualitative manner, the changes that were encountered between the different groups, for each brain structure studied.

**Table 1:** [^3^H]-NFPS binding levels in the rat brain ipsilateral to 6-OHDA injection

	[^3^H]-NFPS binding levels (nCi/mg)			
	Sham	6-OHDA L-DOPA-naïve	6-OHDA Mild AIMs	6-OHDA Severe AIMs
Primary motor cortex	0.158 ± 0.021	0.157 ± 0.028	0.149 ± 0.022	0.146 ± 0.018
Basal ganglia				
Caudate-putamen	0.155 ± 0.011	0.192 ± 0.025*	0.159 ± 0.030	0.168 ± 0.020
Globus pallidus	0.215 ± 0.053	0.234 ± 0.041	0.213 ± 0.025	0.214 ± 0.029
Entopeduncular nucleus	0.142 ± 0.032	0.163 ± 0.019^†††^	0.144 ± 0.017^†††^	0.067 ± 0.115
Subthalamic nucleus	0.119 ± 0.019	0.121 ± 0.025	0.102 ± 0.009^††^	0.129 ± 0.015
Substantia nigra	0.047 ± 0.007	0.052 ± 0.007	0.047 ± 0.005^†^	0.056 ± 0.006
Ventral anterior/ventral lateral thalamus	0.150 ± 0.020	0.115 ± 0.019^&^	0.146 ± 0.021^†††^	0.089 ± 0.010**

Data are presented as the mean ± standard error of the mean (SEM) and are expressed in nCi per mg of tissue

*6-OHDA* 6-hydroxydopamine, *AIMs* abnormal involuntary movements, *L-DOPA* L-3,4-dihydroxyphenylalanine

*: *P* < 0.05, **: *P* < 0.01 compared to sham-lesioned group

^&^: *P* < 0.05 compared to 6-OHDA-lesioned rats with mild AIMs

^†^: *P* < 0.05, ^††^: *P* < 0.01, ^†††^: *P* < 0.001 compared to 6-OHDA-lesioned rats with severe AIMs

**Table 2:** [^3^H]-NFPS binding levels in the rat brain contralateral to 6-OHDA injection

	[^3^H]-NFPS binding levels (nCi/mg)			
	Sham	6-OHDA L-DOPA-naïve	6-OHDA Mild AIMs	6-OHDA Severe AIMs
Primary motor cortex	0.148 ± 0.016	0.164 ± 0.032	0.128 ± 0.011^†^	0.158 ± 0.015
Basal ganglia				
Caudate-putamen	0.197 ± 0.020	0.200 ± 0.024^&^	0.151 ± 0.014	0.175 ± 0.018**
Globus pallidus	0.243 ± 0.039	0.267 ± 0.050	0.246 ± 0.029	0.259 ± 0.037
Entopeduncular nucleus	0.116 ± 0.026	0.126 ± 0.015^†^	0.148 ± 0.014^††^	0.091 ± 0.007
Subthalamic nucleus	0.132 ± 0.022	0.127 ± 0.024	0.111 ± 0.012^†^	0.133 ± 0.018
Substantia nigra	0.045 ± 0.007	0.054 ± 0.011	0.055 ± 0.005	0.055 ± 0.007
Ventral anterior/ventral lateral thalamus	0.137 ± 0.021	0.105 ± 0.020*^†^	0.147 ± 0.025^†††^	0.098 ± 0.009***

Data are presented as the mean ± SEM and are expressed in nCi of GlyT1 per mg of tissue

*6-OHDA* 6-hydroxydopamine, *AIMs* abnormal involuntary movements, *L-DOPA* L-3,4-dihydroxyphenylalanine

*: *P* < 0.05, **: *P* < 0.01, ***: *P* < 0.001 compared to sham-lesioned group

^&^: *P* < 0.05 compared to 6-OHDA-lesioned rats with mild AIMs

^†^: *P* < 0.05, ^††^: *P* < 0.01, ^†††^: *P* < 0.001 compared to 6-OHDA-lesioned rats with severe AIMs

**Table 3:** Qualitative changes in [^3^H]-NFPS binding levels across the different groups in each brain area studied

	[^3^H]-NFPS binding levels							
	6-OHDA L-DOPA-naïve vs. sham		6-OHDA L-DOPA-treated with mild AIMs vs. L-DOPA-naïve		6-OHDA L-DOPA-treated with severe AIMs vs. L-DOPA-naïve		6-OHDA Mild AIMs vs. severe AIMs	
	ipsi	contra	ipsi	contra	ipsi	contra	ipsi	contra
Primary motor cortex	ns	ns	ns	ns	ns	ns	ns	↓
Basal ganglia	ns	ns	ns	ns	ns	ns	ns	ns
Caudate-putamen	↑	ns	ns	↓	ns	ns	ns	ns
Globus pallidus	ns	ns	ns	ns	ns	ns	ns	ns
Entopeduncular nucleus	ns	ns	ns	↓	↓	↓	↑	↑
Subthalamic nucleus	ns	ns	ns	ns	ns	ns	↓	↓
Substantia nigra	ns	ns	ns	ns	ns	ns	↓	ns
Ventral anterior/ventral lateral thalamus	ns	↓	↑	ns	↓	↓	↓	↑

*6-OHDA* 6-hydroxydopamine, *AIMs* abnormal involuntary movements, *L-DOPA* L-3,4-dihydroxyphenylalanine, *ns* not significant

↓: significant reduction in [^3^H]-NFPS binding levels; ↑: significant increase in [^3^H]-NFPS binding levels

#### Specific [^3^H]-NFPS binding in sham-lesioned and L-DOPA-naïve 6-OHDA-lesioned rats

In this section, we provide results that pertain to the effect of 6-OHDA on [^3^H]-NFPS binding. Specific [^3^H]-NFPS binding in the ipsilateral CPu significantly increased in L-DOPA-naïve 6-OHDA-lesioned rats (*t*_(13)_ = 3.877, *P* < 0.05, Fig. 3A) when compared to sham-lesioned ones. Specific [^3^H]-NFPS binding in the contralateral VA/VL thalamus was decreased in L-DOPA-naïve 6-OHDA-lesioned rats when compared to sham-lesioned animals (*t*_(13)_ = 3.483, *P* < 0.05, Fig. 4B).

#### Specific [^3^H]-NFPS binding in L-DOPA-naïve and L-DOPA-treated 6-OHDA-lesioned rats

In this section, we provide results that pertain to the effect of L-DOPA, irrespective of AIMs severity, on [^3^H]-NFPS binding. There was a significant decrease in specific [^3^H]-NFPS binding in the ipsilateral VA/VL thalamus in 6-OHDA-lesioned rats with severe AIMs, compared to sham-lesioned rats (*t*_(15)_ = 4.985, *P* < 0.01, Fig. 3B). Specific [^3^H]-NFPS binding in the ipsilateral VA/VL thalamus significantly increased in 6-OHDA-lesioned rats with mild AIMs, compared to L-DOPA-naïve 6-OHDA lesioned rats (*t*_(12)_ = 3.595, *P* < 0.05, Fig. 3B). There was a significant decrease in specific [^3^H]-NFPS binding in the ipsilateral EPN in 6-OHDA-lesioned rats with severe AIMs, compared to L-DOPA-naïve 6-OHDA lesioned rats (*t*_(7)_ = 9.469, *P* < 0.001, Fig. 3F).

In the hemisphere contralateral to 6-OHDA injection, specific [^3^H]-NFPS binding in the CPu was significantly lower when mildly dyskinetic 6-OHDA-lesioned rats were compared to L-DOPA-naïve 6-OHDA-lesioned rats (*t*_(11)_ = 4.398, *P* < 0.05, Fig. 4A). A reduction of specific [^3^ H]-NFPS binding in the contralateral VA/VL thalamus was also observed in 6-OHDA-lesioned rats with severe AIMs when compared to L-DOPA-naïve 6-OHDA-lesioned rats (*t*_(14)_ = 3.705, *P* < 0.05, Fig. 4B). There was a significant decrease in specific [^3^H]-NFPS binding in the contralateral EPN in 6-OHDA-lesioned rats with severe AIMs, compared to L-DOPA-naïve 6-OHDA lesioned rats (*t*_*(*7)_ = 4.651, *P* < 0.05, Fig. 4F).

#### Specific [^3^H]-NFPS binding in 6-OHDA-lesioned rats with mild and severe AIMs

Specific [^3^H]-NFPS binding in the ipsilateral VA/VL thalamus was significantly decreased in 6-OHDA-lesioned rats with mild AIMs, compared to severely dyskinetic 6-OHDA lesioned rats (*t*_(14)_ = 5.517, *P* < 0.0001, Fig. 3A). There was a significant decrease in specific [^3^H]-NFPS binding in the ipsilateral EPN in 6-OHDA-lesioned rats with severe AIMs, compared to 6-OHDA-lesioned rats with mild AIMs (*t*_(10)_ = 8.909, *P* < 0.001, Fig. 3F). [^3^H]-NFPS binding was significantly lower in the ipsilateral STN and SN in mildly dyskinetic 6-OHDA-lesioned rats when compared to 6-OHDA-lesioned rats with severe AIMs (*t*_(14)_ = 4.739, *P* < 0.01 and *t*_(14)_ = 3.317, *P* < 0.05, Fig. 3D and G, respectively).

In the hemisphere contralateral to injection of 6-OHDA, significant differences were observed in the contralateral VA/VL thalamus between the two AIMs groups, with severely dyskinetic animals presenting lower [^3^H]-NFPS specific binding than mildly dyskinetic rats (*t*_(14)_ = 5.656, *P* < 0.001, Fig. 4B). Differences were also encountered in [^3^H]-NFPS specific binding in the contralateral VA/VL thalamus, which was decreased in severely dyskinetic 6-OHDA-lesioned rats when compared to sham-lesioned animals (*t*_(15)_ = 8.126, *P* < 0.0001, Fig. 4B). There was a significant decrease in specific [^3^H]-NFPS binding in the contralateral EPN in 6-OHDA-lesioned rats with severe AIMs, compared to 6-OHDA-lesioned rats with mild AIMs (*t*_(10)_ = 8.352, *P* < 0.001, Fig. 4F). Specific [^3^H]-NFPS binding in the contralateral M1 and STN was significantly lower in 6-OHDA-lesioned rats with mild AIMs compared to rats with severe AIMs (*t*_(13)_ = 4.126, *P* < 0.05 and *t*_(14)_ = 3.323, *P* < 0.05, Fig. 4C and D, respectively).

### Correlation between [^3^H]-NFPS specific binding and ALO AIMs scores in the 6-OHDA-lesioned rat

**Fig. 5:**
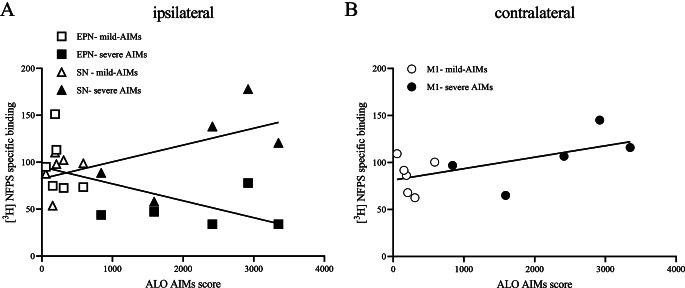
[^3^H]-NFPS binding in the ipsilateral substantia nigra (SN, **A**), and contralateral primary motor cortex (M1, **B**) was positively correlated with mean integrated axial, limbs and oro-lingual (ALO) abnormal involuntary movements (AIMs) scores of L-DOPA-treated 6-OHDA-lesioned animals, while [^3^H]-NFPS binding in the ipsilateral entopeduncular nucleus (EPN, **A**) was negatively correlated. *N* = 5–9 per region per group (for some areas, tissue was missing, which is why 11 points per region are depicted)

As shown in Fig. 5A, there was a correlation between specific [^3^H]-NFPS binding in the SN and EPN ipsilateral to the lesion and ALO AIMs scores of 6-OHDA-lesioned rats (*r* = 0.6269, *P* < 0.05 and *r* = -0.6155, *P* < 0.05, respectively). In addition, specific [^3^H]-NFPS binding density in the M1 contralateral to the injection was positively correlated with ALO AIMs scores of 6-OHDA-lesioned rats (*r* = 0.6024, *P* < 0.05; Fig. 5B). [^3^H]-NFPS specific binding in the other brain regions studied did not correlate with ALO AIMs scores.

As displayed in Figure S1, [^3^H]-NFPS binding in the ipsilateral CPu, M1, STN, GP, and VA/VL thalamus was not correlated with ALO AIMs scores of 6-OHDA-lesioned animals (*r* = 0.2711, *r* = 0.0256, *r* = 0.2676, *r* = 0.0725 and *r* = -0.4855, all *P* > 0.05). In the contralateral CPu, STN, GP, EPN, VA/VL thalamus, and SN of 6-OHDA-lesioned animals, [^3^H]-NFPS binding did not correlate with ALO AIMs scores (*r* = 0.4422, *r* = 0.0559, *r* = 0.2852, *r* = -0.5603, *r* = -0.5293, *r* = 0.2725, all *P* > 0.05; Figure S2).

## Discussion

In this study, we sought to quantify the distribution of GlyT1 in brain areas from the motor loop of the cortex – basal ganglia – thalamus – cortex circuit, which is involved in PD and L-DOPA-induced dyskinesia, in the 6-OHDA-lesioned rat. A key finding of this study was a regionally selective variation of [^3^H]-NFPS binding in 6-OHDA-lesioned rats that exhibited mild or severe dyskinesia. In the VA/VL thalamus, bilateral reductions were observed in severely dyskinetic 6-OHDA-lesioned rats compared to mildly dyskinetic 6-OHDA-lesioned rats. In the EPN, bilateral decreases were observed in severely dyskinetic 6-OHDA-lesioned rats compared to L-DOPA-naïve 6-OHDA-lesioned rats and mildly dyskinetic 6-OHDA-lesioned rats. Ipsilaterally, [^3^H]-NFPS binding was slightly increased in the CPu of L-DOPA-naïve 6-OHDA-lesioned rats and diminished in the STN and SN of 6-OHDA-lesioned rats with mild AIMs compared to 6-OHDA-lesioned animals with severe AIMs. Contralaterally, a decrease of [^3^H]-NFPS binding in the CPu of 6-OHDA-lesioned rats with mild AIMs was observed compared to sham-lesioned and L-DOPA-naïve 6-OHDA-lesioned animals. Reductions of [^3^H]-NFPS binding were also demonstrated in M1 and STN of 6-OHDA-lesioned rats with mild AIMs compared to those with severe AIMs. In contrast, [^3^H]-NFPS specific binding remained unchanged in the ipsilateral M1, contralateral SN and bilateral GP between 6-OHDA-lesioned rats and sham-lesioned ones. Additionally, dyskinesia severity was positively correlated with [^3^H]-NFPS binding levels in the ipsilateral SN and contralateral M1 and negatively correlated with [^3^H]-NFPS binding levels in the ipsilateral EPN. Overall, these findings suggest that GlyT1 is implicated in the pathophysiology of PD and L-DOPA induced dyskinesia.

To determine GlyT1 levels, we have used NFPS, a potent and selective sarcosine-based GlyT1 inhibitor with high affinity for its target (dissociation constant [Kd] of 7.1 nM) (Mallorga et al. 2003) that binds at a different site than glycine (Zeng et al. 2008; Herdon et al. 2010). The cold ligand employed, Org-25,935, is another sarcosine-derived GlyT1 inhibitor that harbours high selectivity and affinity to its target (half-maximal inhibitory concentration [IC_50_] of 100 nM). The concentration employed here, 1 µM, was thus well above Org-25,935 IC_50_ at GlyT1, preventing [^3^H]-NFPS to bind to the transporter.

There is one caveat that we would like to emphasise, which is the inclusion of female rats only, which prevents our results from being generalised to male individuals. We acknowledge this limitation of our study and urge the readers to keep it in mind while reading the paragraphs below. Encouragingly, in a study conducted with ALX-5407 in the MPTP-lesioned marmoset, we did not observe any difference between female and male animals (Frouni et al. 2021), suggesting that sex-related differences may be minimal.

In the present study, the reduction of TH optical density in the striatum was of lesser magnitude in the group of 6-OHDA-lesioned rats with mild AIMs than in the group with severe AIMs. However, this difference was not statistically significant, which indicates that the extent of dopaminergic denervation was similar in both groups, which suggests that different degrees of denervation do not explain the difference in dyskinesia severity between the groups we obtained here. We would like to mention that it is well established in the literature that a subset of L-DOPA-treated 6-OHDA-lesioned rats do not develop dyskinesia, or develop mild dyskinesia, independently of the degree of denervation (Hamadjida et al. 2019).

In sham-lesioned animals, [^3^H]-NFPS binding was highest in the GP, the M1, the CPu, and the thalamus, with intermediate levels in the STN and low levels in the SN. A previous study had evaluated GlyT1 distribution in healthy rats, using [^3^H]-GSK931145 as the radioligand and discovered higher binding in the thalamus, cerebellum, pons and medulla (Herdon et al. 2010). In our current experiments, we did not look at structures beyond the cortex – basal ganglia – thalamus – cortex circuit, and therefore did not quantify GlyT1 density in the cerebellum and brain stem, so no comparison can be made, but the high levels encountered in the thalamus would suggest that our results are in agreement with this previous study. Another study using [^18^F]-ALX-5407 as the radioligand similarly discovered high GlyT1 densities in the thalamus with moderate levels in the CPu in healthy rats (Hoffmann et al. 2021). As this is the first study to quantify the distribution of GlyT1 in 6-OHDA lesioned rats, it is not possible to compare our findings with previous experiments.

In hemi-parkinsonian rats that were not exposed to L-DOPA, we discovered lower GlyT1 levels in the contralateral thalamus compared to control animals. We propose that this reduction of GlyT1 density in the thalamus is part of a compensatory mechanism in parkinsonism. Thus, reduced GlyT1 in the thalamus would result in higher synaptic glycine levels and, potentially, enhancing glutamatergic transmission at *N*-methyl-D-aspartate (NMDA) receptors, as glycine is a co-agonist at NMDA receptors (Danysz and Parsons 1998). According to the model of the organisation of the basal ganglia (McGregor and Nelson 2019), activation of NMDA receptors in the thalamus would result in greater cortical activation, which would have a facilitatory effect on movement. Admittedly, this compensatory mechanism would be only partly efficacious, as the animals in our experiments were severely parkinsonian. Accordingly, it is possible that the anti-parkinsonian benefit we obtained in 6-OHDA-lesioned rats with bitopertin (Frouni et al. 2022) and in MPTP-lesioned marmosets with ALX-5407 (Frouni et al. 2021) was mediated through an activation of the thalamo-cortical pathway.

Similar to the L-DOPA-naïve parkinsonian state, in 6-OHDA-lesioned rats with severe dyskinesia, we also found lower GlyT1 levels in the thalamus compared to sham-lesioned animals. In contrast to the hypothesis put forth in the previous paragraph regarding a possible compensatory mechanism of reduced GlyT1 in the thalamus, it is possible that this reduction in thalamic GlyT1 might contribute to the dyskinetic phenotype, as it would ultimately result in higher cortical activation. Then, how can these findings be reconciled with an anti-dyskinetic effect of GlyT1 inhibition in both the 6-OHDA-lesioned rat (Frouni et al. 2022) and the MPTP-lesioned primate (Frouni et al. 2021)? When present in higher concentrations within the synapse, glycine may lead to NMDA receptor internalisation (Nong et al. 2003), which would result in decreased glutamatergic transmission; along the thalamo-cortical pathway, it would lead to decreased cortical activation, and less abnormal movements. Ultimately, the implication is that GlyT1 inhibition would normalise thalamo-cortical transmission in such a manner that it might both alleviate parkinsonism and dyskinesia. It should be mentioned however, that GlyT1 inhibitors might act at other structures along the cortex – basal ganglia – thalamus – cortex. Studies where intra-cerebral injections would be performed with GlyT1 inhibitors, would be key to further define the role of the brain areas in which we encountered differences in GlyT1 binding levels.

As for the mildly dyskinetic state, a decrease in GlyT1 levels was observed in the STN, coupled with an increase in the thalamus, in comparison to the severely dyskinetic state. We propose that this may be part of an endogenous compensatory mechanism, which perhaps could explain why some rats did not develop AIMs as severe as others, despite being severely lesioned and administered the same L-DOPA regimen. According to the model of the basal ganglia, increased glycine levels in the STN would result in enhanced activation of the output structures of the basal ganglia, the EPN and the SN pars reticulata, leading to greater inhibition of the thalamus and less cortical activation, diminishing overall movement. For reasons yet to be identified, only a subset of 6-OHDA-lesioned rats would develop this compensatory mechanism. Of note, by the same token, an action at the STN, although speculative, might be a mechanism whereby GlyT1 inhibition might alleviate dyskinesia. Regarding the thalamus, we note that GlyT1 levels, while lower than those found in severely dyskinetic rats, were not different to those encountered in sham-lesioned animals, which would suggest a possible compensatory attempt by the brain to normalise thalamo-cortical transmission.

In summary, we have discovered that GlyT1 density is altered in the thalamus in non-dyskinetic and dyskinetic hemi-parkinsonian rats, possibly representing compensatory attempts by the brain to normalise the altered neurochemistry of the brain in parkinsonism and dyskinesia. We also suggested that the STN might also be part of this compensatory effort. We have also tried to speculate on how these alterations in GlyT1 density might fit with the therapeutic effects of GlyT1 inhibition on both parkinsonian disability and dyskinesias in pre-clinical models of PD. We acknowledge that our study has limitations, but we nevertheless believe that it refines our comprehension of the cortex – basal ganglia – thalamus – cortex in the parkinsonian and dyskinetic states, and improves our understanding of how GlyT1 inhibitors might elicit their therapeutic benefits.

### Electronic supplementary material

Below is the link to the electronic supplementary material.

Supplementary Material 1

## Data Availability

Additional data are available as Supplementary Materials. Raw data will be made available upon request to the Corresponding Author.

## References

[CR1] Borroni E, Zhou Y, Ostrowitzki S (2013). Pre-clinical characterization of [11 C] R05013853 as a novel radiotracer for imaging of the glycine transporter type 1 by positron emission tomography. NeuroImage.

[CR2] Cenci MA, Lundblad M (2007). Ratings of L-DOPA-induced dyskinesia in the unilateral 6-OHDA lesion model of Parkinson’s disease in rats and mice. Curr Protoc Neurosci Chap 9:Unit.

[CR3] Cubelos B, Giménez C, Zafra F (2005). Localization of the GLYT1 glycine transporter at glutamatergic synapses in the rat brain. Cereb Cortex.

[CR4] Danysz W, Parsons CG (1998). Glycine and N-methyl-D-aspartate receptors: physiological significance and possible therapeutic applications. Pharmacol Rev.

[CR6] Fox SH, Lang AE (2008). Levodopa-related motor complications—phenomenology. Mov Disorders: Official J Mov Disorder Soc.

[CR5] Fox SH, Katzenschlager R, Lim SY (2018). International Parkinson and movement disorder society evidence-based medicine review: update on treatments for the motor symptoms of Parkinson’s disease. Mov Disord.

[CR10] Frouni I, Kwan C, Bedard D (2018). Effect of the selective 5-HT2A receptor antagonist EMD-281,014 on L-DOPA-induced abnormal involuntary movements in the 6-OHDA-lesioned rat. Exp Brain Res.

[CR8] Frouni I, Hamadjida A, Kwan C (2019). Activation of mGlu2/3 receptors, a novel therapeutic approach to alleviate dyskinesia and psychosis in experimental parkinsonism. Neuropharmacology.

[CR7] Frouni I, Belliveau S, Maddaford S, Nuara SG, Gourdon JC, Huot P (2021). Effect of the glycine transporter 1 inhibitor ALX-5407 on dyskinesia, psychosis-like behaviours and parkinsonism in the MPTP-lesioned marmoset. Eur J Pharmacol.

[CR9] Frouni I, Kang W, Bédard D (2022). Effect of glycine transporter 1 inhibition with bitopertin on parkinsonism and L-DOPA induced dyskinesia in the 6-OHDA-lesioned rat. Eur J Pharmacol.

[CR11] Hamadjida A, Frouni I, Kwan C, Huot P (2019). Classic animal models of Parkinson’s disease: a historical perspective. Behav Pharmacol.

[CR12] Hamadjida A, Sid-Otmane L, Kwan C (2020). The highly selective mGlu2 receptor positive allosteric modulator LY‐487,379 alleviates l‐DOPA‐induced dyskinesia in the 6‐OHDA‐lesioned rat model of Parkinson’s disease. Eur J Neurosci.

[CR13] Hely MA, Morris JG, Reid WG, Trafficante R (2005). Sydney multicenter study of Parkinson’s disease: Non-L‐dopa–responsive problems dominate at 15 years. Mov Disorders: Official J Mov Disorder Soc.

[CR14] Herdon HJ, Roberts JC, Coulton S, Porter RA (2010). Pharmacological characterisation of the GlyT-1 glycine transporter using two novel radioligands. Neuropharmacology.

[CR15] Hoffmann C, Evcüman S, Neumaier F (2021). [18F] ALX5406: a brain-penetrating Prodrug for GlyT1-Specific PET imaging. ACS Chem Neurosci.

[CR16] Hornykiewicz O, Kish S (1987). Biochemical pathophysiology of Parkinson’s disease. Adv Neurol.

[CR17] Kwan C, Lévesque C, Bédard D (2022). Autoradiographic labelling of 5-HT3 receptors in the hemi-parkinsonian rat brain. Neurosci Res.

[CR19] Mallorga PJ, Williams JB, Jacobson M (2003). Pharmacology and expression analysis of glycine transporter GlyT1 with [3H]-(N-[3-(4′-fluorophenyl)-3-(4′ phenylphenoxy) propyl]) sarcosine. Neuropharmacology.

[CR20] McGregor MM, Nelson AB (2019). Circuit mechanisms of Parkinson’s disease. Neuron.

[CR21] Nong Y, Huang Y-Q, Ju W, Kalia LV, Ahmadian G, Wang YT, Salter MW (2003). Glycine binding primes NMDA receptor internalization. Nature.

[CR24] Paxinos G, Watson C (2007). The rat brain in stereotaxic coordinates.

[CR25] Schallert T, Fleming SM, Leasure JL, Tillerson JL, Bland ST (2000). CNS plasticity and assessment of forelimb sensorimotor outcome in unilateral rat models of stroke, cortical ablation, parkinsonism and spinal cord injury. Neuropharmacology.

[CR26] Schmitz Y, Castagna C, Mrejeru A, Lizardi-Ortiz JE, Klein Z, Lindsley CW, Sulzer D (2013). Glycine transporter-1 inhibition promotes striatal axon sprouting via NMDA receptors in dopamine neurons. J Neurosci.

[CR27] Ungerstedt U (1968). 6-Hydroxy-dopamine induced degeneration of central monoamine neurons. Eur J Pharmacol.

[CR28] Wong DF, Ostrowitzki S, Zhou Y (2013). Characterization of [11 C] RO5013853, a novel PET tracer for the glycine transporter type 1 (GlyT1) in humans. NeuroImage.

[CR29] Zafra F, Gomeza J, Olivares L, Aragón C, Giménez C (1995). Regional distribution and developmental variation of the glycine transporters GLYT1 and GLYT2 in the rat CNS. Eur J Neurosci.

[CR30] Zeng Z, O’Brien JA, Lemaire W (2008). A novel radioligand for glycine transporter 1: characterization and use in autoradiographic and in vivo brain occupancy studies. Nucl Med Biol.

[CR31] Zilles K, Palomero-Gallagher N, Grefkes C, Scheperjans F, Boy C, Amunts K, Schleicher A (2002). Architectonics of the human cerebral cortex and transmitter receptor fingerprints: reconciling functional neuroanatomy and neurochemistry. Eur Neuropsychopharmacol.

