# Supplementary material for: [3H]-NFPS binding to the glycine transporter 1 in the hemi-parkinsonian rat brain
Source: Exp Brain Res. 2024 Mar 25;242(5):1203–14. doi: 10.1007/s00221-024-06815-w (PMC11078860; doi:10.1007/s00221-024-06815-w)
Supplement: Supplementary file 1 — Supplementary Material 1 [file 221_2024_6815_MOESM1_ESM.docx]

**Table S1.** Summary of multiple Student *t* tests for [^3^H]-NFPS binding levels ipsilateral to 6-OHDA injection

|  | group comparisons | degrees of freedom | *t* ratio | *P* value |
| --- | --- | --- | --- | --- |
| primary motor cortex | sham – L-DOPA naïve | 13 | 0.01252 | 0.9028 |
|  | sham – mild AIMs | 12 | 0.7255 | 0.9807 |
|  | sham – severe AIMs | 15 | 1.0840 | 0.7709 |
|  | mild AIMs – severe AIMs | 13 | 0.1670 | 0.9978 |
| **basal ganglia** |  |  |  |  |
| caudate-putamen | sham – L-DOPA naïve | 13 | 3.8770 | **0.0264*** |
|  | sham – mild AIMs | 12 | 0.3390 | 0.9988 |
|  | sham – severe AIMs | 15 | 1.6910 | 0.5626 |
|  | mild AIMs – severe AIMs | 13 | 0.7264 | 0.9622 |
| globus pallidus | sham – L-DOPA naïve | 9 | 0.6654 | 0.9877 |
|  | sham – mild AIMs | 9 | 0.0686 | 0.9997 |
|  | sham – severe AIMs | 12 | 0.0648 | 0.9944 |
|  | mild AIMs – severe AIMs | 13 | 0.0176 | 0.9978 |
| entopeduncular nucleus | sham – L-DOPA naïve | 7 | 1.1490 | 0.9343 |
|  | sham – mild AIMs | 10 | 0.1428 | 0.9997 |
|  | sham – severe AIMs | 8 | 4.9630 | **0.0132*** |
|  | mild AIMs – severe AIMs | 10 | 8.9090 | **0.0001**^†††^ |
| subthalamic nucleus | sham – L-DOPA naïve | 13 | 0.2764 | 0.9903 |
|  | sham – mild AIMs | 13 | 2.6630 | 0.1873 |
|  | sham – severe AIMs | 15 | 1.1830 | 0.7709 |
|  | mild AIMs – severe AIMs | 14 | 4.7390 | **0.0032**^††^ |
| substantia nigra | sham – L-DOPA naïve | 11 | 1.9230 | 0.5693 |
|  | sham – mild AIMs | 11 | 0.0175 | 0.9997 |
|  | sham – severe AIMs | 13 | 2.6800 | 0.1893 |
|  | mild AIMs – severe AIMs | 14 | 3.3170 | **0.0395**^†^ |
| ventral anterior/ ventral lateral thalamus | sham – L-DOPA naïve | 13 | 3.0850 | 0.0995 |
|  | sham – mild AIMs | 13 | 0.8433 | 0.9764 |
|  | sham – severe AIMs | 15 | 4.9850 | **0.0021**** |
|  | mild AIMs – severe AIMs | 14 | 5.5170 | **0.0008**^†††^ |

Paired *t* tests were performed for each region of interest. To correct for multiple Student *t* tests, the Holm-Sidak correction was applied to adjust *P* values. AIMs, abnormal involuntary movements; L-DOPA, L-3,4-dihydroxyphenylalanine. *N*=5-9 per group. *: *P* < 0.05, **: *P* < 0.01 compared to sham-lesioned group; ^†^: *P* < 0.05, ^††^:*P* < 0.01 and ^†††^: *P* < 0.001 compared to mild AIMs 6-OHDA-lesioned.

**Table S2.** Summary of multiple Student *t* tests for [^3^H]-NFPS binding levels contralateral to 6-OHDA injection

|  | group comparisons | degrees of freedom | *t* ratio | *P* value |
| --- | --- | --- | --- | --- |
| primary motor cortex | sham – L-DOPA naïve | 13 | 1.2510 | 0.9080 |
|  | sham – mild AIMs | 12 | 2.7090 | 0.1873 |
|  | sham – severe AIMs | 15 | 1.3410 | 0.7379 |
|  | mild AIMs – severe AIMs | 13 | 4.1260 | **0.0107**^†^ |
| **basal ganglia** |  |  |  |  |
| caudate-putamen | sham – L-DOPA naïve | 13 | 0.2403 | 0.9903 |
|  | sham – mild AIMs | 12 | 4.8070 | **0.0060**** |
|  | sham – severe AIMs | 15 | 2.4220 | 0.2297 |
|  | mild AIMs – severe AIMs | 13 | 2.7780 | 0.0904 |
| globus pallidus | sham – L-DOPA naïve | 9 | 0.8610 | 0.9756 |
|  | sham – mild AIMs | 9 | 8.3820 | 0.9997 |
|  | sham – severe AIMs | 12 | 0.7636 | 0.8424 |
|  | mild AIMs – severe AIMs | 13 | 0.7012 | 0.9622 |
| entopeduncular nucleus | sham – L-DOPA naïve | 7 | 0.6779 | 0.9877 |
|  | sham – mild AIMs | 10 | 2.8040 | 0.1873 |
|  | sham – severe AIMs | 8 | 2.1370 | 0.4164 |
|  | mild AIMs – severe AIMs | 10 | 8.3520 | **0.0001**^†††^ |
| subthalamic nucleus | sham – L-DOPA naïve | 13 | 0.5086 | 0.9877 |
|  | sham – mild AIMs | 13 | 2.7380 | 0.1852 |
|  | sham – severe AIMs | 15 | 0.0957 | 0.9944 |
|  | mild AIMs – severe AIMs | 14 | 3.3230 | **0.0395**^†^ |
| substantia nigra | sham – L-DOPA naïve | 11 | 2.7980 | 0.1749 |
|  | sham – mild AIMs | 11 | 2.9210 | 0.1665 |
|  | sham – severe AIMs | 13 | 2.6500 | 0.1893 |
|  | mild AIMs – severe AIMs | 14 | 0.0843 | 0.9978 |
| ventral anterior/ ventral lateral thalamus | sham – L-DOPA naïve | 13 | 3.4830 | 0.0513 |
|  | sham – mild AIMs | 13 | 1.509 | 0.7405 |
|  | sham – severe AIMs | 15 | 8.1260 | **0.00001***** |
|  | mild AIMs – severe AIMs | 14 | 5.656 | **0.0007**^†††^ |

Paired *t* tests were performed for each region of interest. To correct for multiple Student *t* tests, the Holm-Sidak correction was applied to adjust *P* values. AIMs, abnormal involuntary movements; L-DOPA, L-3,4-dihydroxyphenylalanine. *N*=5-9 per group. **: *P* < 0.01, ***: *P* < 0.001 compared to sham-lesioned group. †: *P* < 0.05, †††: *P* < 0.001 compared to mild AIMs 6-OHDA-lesioned group.

# Supplementary Figure legends

**Supplementary Figure 1: No correlation between mean integrated ALO AIMs scores and [^3^H]-NFPS binding levels in the ipsilateral** **caudate-putamen, primary motor cortex, subthalamic nucleus, globus pallidus, and ventral anterior/ ventral lateral thalamus.**

[^3^H]-NFPS binding in the ipsilateral caudate-putamen (CPu, A), primary motor cortex (M1, B), subthalamic nucleus (STN, C), globus pallidus (GP, D), and thalamus (E) was not correlated with mean ALO AIMs scores of 6-OHDA-lesioned animals. 6-OHDA, 6-hydroxydopamine; AIMs, abnormal involuntary movements; ALO, axial, limbs and oro-lingual. *N*=11.

**Supplementary Figure 2: No correlation between mean integrated ALO AIMs scores and [^3^H]-NFPS binding levels in the contralateral** **caudate-putamen, subthalamic nucleus, globus pallidus, entopeduncular nucleus, ventral anterior/ ventral lateral thalamus, and substantia nigra.**

Similarly, binding in the contralateral caudate-putamen (CPu, A), subthalamic nucleus (STN, B), globus pallidus (GP, C), entopeduncular nucleus (EPN, D), thalamus (E), and substantia nigra (SN, F) of 6-OHDA-lesioned animals did not correlate with ALO AIMs scores. 6-OHDA, 6-hydroxydopamine; AIMs, abnormal involuntary movements; ALO, axial, limbs and oro-lingual. *N*=11.

# Supplementary Figure 1

# Supplementary Figure 2
